# Lower Respiratory Tract Infection and Short-Term Outcome in Patients With Acute Respiratory Distress Syndrome

**DOI:** 10.1177/0885066618772498

**Published:** 2018-04-26

**Authors:** Fernando G. Zampieri, Pedro Póvoa, Jorge I. Salluh, Alejandro Rodriguez, Sandrine Valade, José Andrade Gomes, Jean Reignier, Elena Molinos, Jordi Almirall, Nicolas Boussekey, Lorenzo Socias, Paula Ramirez, William N. Viana, Anahita Rouzé, Saad Nseir, Ignacio Martin-Loeches

**Affiliations:** 1 HC or Research Institute, Hospital do Coração, São Paulo, Brazil; 2 Polyvalent Intensive Care Unit, São Francisco Xavier Hospital, Centro Hospitalar de Lisboa Ocidental, Lisbon, Portugal; 3 Nova Medical School, CEDOC, New University of Lisbon, Lisbon, Portugal; 4 Department of Critical Care and Graduate Program in Translational Medicine, D’Or Institute for Research and Education, Rio de Janeiro, Brazil; 5 Programa de Pós-graduação em Clínica Médica, Universidade Federal do Rio de Janeiro, Rio Janeiro, Brazil; 6 Critical Care Department, Hospital Universitario Joan XXIII, URV/IISPV/CIBERES, Terragona, Spain; 7 Intensive Care Unit, Saint-Louis University Hospital, AP-HP and Paris-Diderot University, Sorbonne Paris Cité, Paris, France; 8 Unidade de Cuidados Intensivos, Hospital da Luz Lisboa, Lisboa, Portugal; 9 Medical ICU, CHU de Nantes, Nantes, France; 10 Serviço de Medicina Intensiva, Hospital Pedro Hispano, ULS de Matosinhos, Matosinhos, Portugal; 11 Servei de Medicina Intensiva, Hospital de Mataró, Mataró, Barcelona, España; Centro de Investigación Biomédica en Red de Enfermedades Respitarorias (CIBERES), Universitat Autònoma de Barcelona, Barcelona, España; 12 Service de Réanimation Médicale et Maladies Infectieuses, Hôpital Chatiliez, Tourcoing, France; 13 Critical Care Department, Hospital Son Llatzer, Carretera Manacor, Mallorca, Spain; 14 Critical Care Department, Hospital La Fe, Valencia, Spain; 15 Hospital Copa D’Or, Copacabana, Rio de Janeiro, Brazil; 16 Critical Care Center, University Hospital of Lille, Lille University, Lille, France; 17 Multidisciplinary Intensive Care, St James’s University Hospital, Dublin, Ireland; 18 Department of Clinical Medicine, Trinity College, Dublin, Ireland; 19 Welcome Trust-HRB Clinical Research Facility, St James Hospital, Dublin, Ireland

**Keywords:** acute respiratory distress syndrome, ventilator-associated pneumonia, critical care

## Abstract

**Objective:**

To assess whether ventilator-associated lower respiratory tract infections (VA-LRTIs) are associated with mortality in critically ill patients with acute respiratory distress syndrome (ARDS).

**Materials and Methods:**

Post hoc analysis of prospective cohort study including mechanically ventilated patients from a multicenter prospective observational study (TAVeM study); VA-LRTI was defined as either ventilator-associated tracheobronchitis (VAT) or ventilator-associated pneumonia (VAP) based on clinical criteria and microbiological confirmation. Association between intensive care unit (ICU) mortality in patients having ARDS with and without VA-LRTI was assessed through logistic regression controlling for relevant confounders. Association between VA-LRTI and duration of mechanical ventilation and ICU stay was assessed through competing risk analysis. Contribution of VA-LRTI to a mortality model over time was assessed through sequential random forest models.

**Results:**

The cohort included 2960 patients of which 524 fulfilled criteria for ARDS; 21% had VA-LRTI (VAT = 10.3% and VAP = 10.7%). After controlling for illness severity and baseline health status, we could not find an association between VA-LRTI and ICU mortality (odds ratio: 1.07; 95% confidence interval: 0.62-1.83; P = .796); VA-LRTI was also not associated with prolonged ICU length of stay or duration of mechanical ventilation. The relative contribution of VA-LRTI to the random forest mortality model remained constant during time. The attributable VA-LRTI mortality for ARDS was higher than the attributable mortality for VA-LRTI alone.

**Conclusion:**

After controlling for relevant confounders, we could not find an association between occurrence of VA-LRTI and ICU mortality in patients with ARDS.

## Introduction

Acute respiratory distress syndrome (ARDS) is a common and severe condition occurring in the most severely ill patients admitted to the intensive care unit (ICU).^1^ The current consensus as well as older definitions all demand that oxygenation impairment coupled with bilateral opacities on chest imaging in the absence of signs of fluid overload must be present to consider diagnosing ARDS.^1,2^

Lower respiratory tract infections (LRTIs) are a major issue in critically ill patients, being associated with prolonged hospitalization, higher costs, and possibly, an increase in mortality.^3–6^ In mechanically ventilated patients, ventilator-associated LRTIs (VA-LRTIs) are stratified into ventilator-associated tracheobronchitis (VAT) and ventilator-associated pneumonia (VAP) according to the presence of new abnormal chest imaging findings, which are a prerequisite for the diagnosis of VAP.^3^ Since VAP may impair oxygenation, there is, therefore, an important interplay between ARDS and VAP, which challenges both diagnoses in clinical practice. Moreover, the impact of the occurrence of VA-LRTI in patients with ARDS on mortality is debatable, with most reports focusing on VAP only.^4,6–8^

We hypothesized that, in the context of a high mortality scenario such as ARDS, occurrence of VA-LRTI would not be associated with increased ICU mortality. We secondarily hypothesized that the attributable mortality in mechanically ventilated patients would be higher for ARDS than for VA-LRTI alone.

## Materials and Methods

### Population

This is a subanalysis of a large prospective cohort of 2960 critically ill patients, which required mechanical ventilation (MV) for more than 48 hours in 8 countries (incidence and prognosis of ventilator-associated tracheobronchitis—TAVeM study^3^). According to the Berlin definition, all patients with ARDS were selected for this subanalysis.^1^ A full list of investigators is given in the Supplemental Material.

### Definitions

Only microbiologically confirmed infections were considered for this analysis.^3^ A diagnosis of VA-LRTI was made when 2 of the following criteria were present: temperature > 38.5°C or < 36.5°C, leukocyte count greater than 12 000 cells/μL or less than 4000 cells/μL, and purulent secretions on tracheal aspirate. Ventilator-associated lower respiratory tract infection was stratified into VAT or VAP according to the presence or absence of new abnormal imaging on chest radiography; patients without new infiltrates were categorized as having VAT, while patients with new infiltrates were considered to have VAP.^3^ All chest radiographies were centrally adjudicated by members of the steering committee.^3^ We only included the first episodes of VA-LRTI occurring more than 48 hours after starting MV. The diagnosis of ARDS was made based on the Berlin criteria using Pao_2_/Fio_2_ (P/F) ratio^9^ at the time the patient was included in the main study; therefore, all patients had ARDS prior to development of VA-LRTI.

### End Points

The main end point was ICU mortality. Secondary end points included ICU length of stay (LOS) and length of MV.

### General Statistical Analysis

Continuous variables were compared between groups with *t* test or Mann-Whitney *U* test according to the normality assessment using Kolmogorov-Smirnov test. Categorical variables were compared using χ^2^ test or Fisher exact test, as appropriate.

### Mortality Analysis

The independent association between VA-LRTI and ICU mortality in patients with ARDS was assessed using logistic regression model. We defined a priori that the model would adjust for a marker of global illness severity (Simplified Acute Physiology Score 2 [SAPS 2]^10^), a marker of performance in daily living activities (Barthel index^11^), worsening in gas oxygenation, and occurrence of VA-LRTI. Two main models were built, one grouping VAT and VAP (VA-LRTI model) and one considering VAT and VAP individually while coded as dummy variables. Models were bootstrapped (10^5^ repetitions) and bootstrap bias reported.

### Attributable Mortality Analysis

For attributable mortality analysis, we elected all patients included in the TAVeM database. A multivariate logistic regression with ICU mortality as the dependent variable was performed with the following predictors: SAPS 2, Barthel index, occurrence of ARDS, and occurrence of VA-LRTI. Interaction between occurrence of ARDS and VA-LRTI was allowed in the model. We estimated the attributable fraction under the hypothetical scenario where the binary exposure of interest (ARDS or VA-LRTI) is eliminated from the population based on a generalized linear model (ratio of counterfactual probabilities). We calculated attributable mortality of VA-LRTI in patients with and without ARDS. In a sensitivity analysis, we considered only VAP instead of VA-LRTI for attributable mortality estimation.

### Contribution of VA-LRTI to Mortality Over Time

We performed a secondary mortality analysis using sequential random forests models to assess the impact of timing of occurrence of VA-LRTI on mortality in both patients with and without ARDS. In this analysis, random forest models were built for each day from the first day of follow-up until 14 days. Random forest models are tree-based classifying methods that are useful for classification when interactions are expected and that provide reliable estimators of variable importance.^12,13^ For each day, the random forest model was performed including only patients who were still in the ICU up to that day and who did not have VA-LRTI up to that day. For example, the random forest model of day 5 included only patients who were still in the ICU at that day and who did not have VA-LRTI until then. The model was adjusted for SAPS 2 and Barthel index. We plotted the relative importance of the 3 variables included in the model (SAPS 2, Barthel index, and VA-LRTI) over time. Variable importance was defined as the mean decrease in Gini score due to that variable in the model, which is presented as percentage over all Gini decrease for clarity. Gini importance measures the average gain of purity of splits in a random forest model; the more useful a variable is, the higher the “purity” in the nodes after splitting (ie, the more importance a variable is, the higher will be the decrease in Gini score caused by it). This analysis is conceptually similar to the one performed by Iwashyna.^14^ We truncated this analysis at 14 days to the limited number of events after the period.

### Intensive Care Unit LOS and Duration of MV

The association between ICU LOS and duration of MV and VA-LRTI was assessed using a Fine and Grey–adjusted competing risk models considering death as a competitor for ICU LOS and duration of MV. Similar to the logistic regression, the model was adjusted by SAPS 2, Barthel index, and worsening of oxygenation.

All analyses were performed using R project version 3.4.0 with packages tidyverse, randomForest, ggplot2, and AF.^15^ A *P* value of .05 was considered significant for the analyses except when mentioned.

## Results

**Figure 1.**
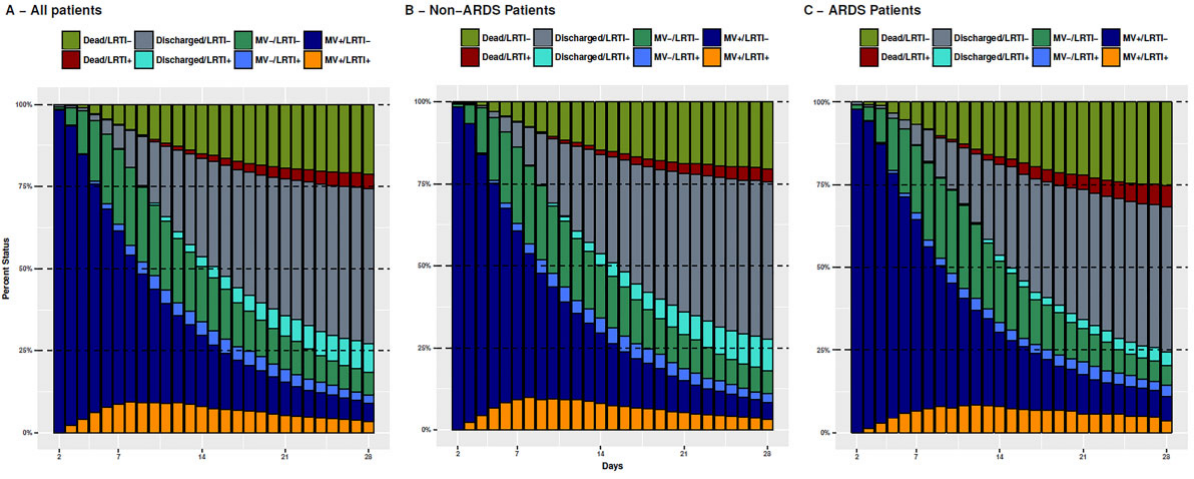
Proportion of patients at a given status from admission until 28 days (A), stratified according to the absence (B) or presence (C) of acute respiratory distress syndrome (ARDS). There are 4 possible status (on mechanical ventilation: MV[+]; not on MV but still in the intensive care unit [ICU]: MV[−]; and discharged alive from the ICU or dead) subsequently divided according to the presence or absence of ventilator-associated lower respiratory tract infection (VA-LRTI) in the patient (LRTI [+] or LRTI[−], respectively). Note the slightly higher mortality for patients having ARDS without (green) or with (red) previous VA-LRTI. There was a smaller proportion of patients discharged after VA-LRTI in the ARDS subgroup (cyan bars).

Of the initial cohort of 2960 patients, 524 fulfilled Berlin definition for ARDS. The time course of patients in the TAVeM database is shown in Figure 1, with sequential 100% stacked barplots representing the proportion of patients on a given status at a given day; panels B and C show the information for patients with and without ARDS. Patients were categorized according to the clinical status (at the ICU on MV, at the ICU not on MV, and discharged or dead) stratified by the presence of VA-LRTI up to that day (see Figure 1 for details). Time trend of patients with and without ARDS was similar, with some particularities such as the higher mortality in patients with ARDS (both in patients with [red color] or without [dark green color] LRTI) and the reduced proportion of patients discharged alive after VA-LRTI in the ARDS group (noticed by the reduced cyan area). These differences, however, were small.

**Figure 2.**
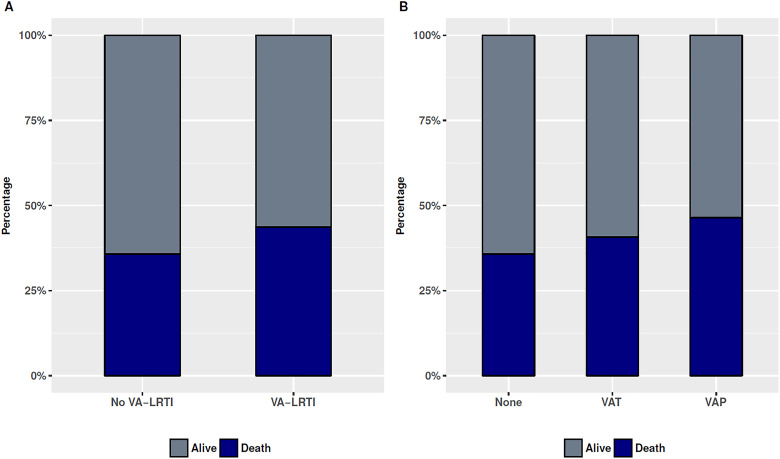
Intensive care unit (ICU) mortality stratified according to (A) the presence or absence of ventilator-associated lower respiratory tract infection (VA-LRTI) or (B) occurrence of ventilator-associated tracheobronchitis (VAT), ventilator-associated pneumonia (VAP), or no-LRTI.

Patients with ARDS were more frequently admitted due to medical reasons, had higher Sequential Organ Failure Assessment score, more comorbid conditions (chronic obstructive pulmonary disease, diabetes, alcoholism, and hematological cancer), and had a higher ICU mortality. The occurrence of microbiologically confirmed VAT and VAP was not different between patients with and without ARDS. A comparison between patients with and without ARDS is shown in Supplemental Table 1. Patients with ARDS who died in the ICU were older, had higher illness severity, and more frequently had cancer (Table 1). Although ICU mortality was higher for patients having ARDS with VAT or VAP (VAT 22 [40.7%] of 54 and VAP 26 [46.4%] of 56; Figure 2), this did not reach statistical significance (*P* =.261). The results were unchanged if VAT/VAP was grouped in VA-LRTI (mortality 48 [43.6%] of 110 vs 148 [35.7%] 414 in patients having ARDS without VA-LRTI; *P* =.158).

**Table 1.** Comparison Between Survivor and Nonsurvivor Patients With ARDS.

Characteristics	Survivors	Death	*P*
Number of patients	328	196	
Age, mean (SD)	60.3 (16.5)	64.2 (5.4)	.07
Male gender, n (%)	192 (58.5)	118 (60.2)	.77
Admission type, n (%)			.77
Medical	256 (78.0)	148 (75.5)	
Surgical	54 (16.5)	37 (18.9)	
Trauma	18 (5.5)	11 (5.6)	
SAPS 2, mean (SD)	48.2 (18.7)	55.9 (18.6)	<.001
Barthel, mean (SD)	85.4 (28.3)	81.3 (29.4)	.11
SOFA, mean (SD)	8.4 (3.9)	9.6 (4.0)	.001
COPD, n (%)	45 (13.7)	26 (13.3)	.99
Chronic renal failure, n (%)	40 (12.2)	33 (16.8)	.18
Diabetes, n (%)	69 (21.0)	53 (27.0)	.14
Alcoholism, n (%)	28 (8.5)	19 (9.7)	.77
Nonmetastatic cancer, n (%)	28 (8.5)	29 (14.8)	.04
Metastatic cancer, n (%)	5 (1.5)	10 (5.1)	.03
Hematologic cancer, n (%)	12 (3.7)	24 (12.2)	<.001
AIDS, n (%)	2 (0.6)	5 (2.6)	.14
Worsening X-ray, n (%)	42 (12.8)	33 (16.8)	.25
Worsening gas exchange, n (%)	48 (14.6)	42 (21.4)	.06
Time to infection, median (IQR)	7.0 (4.0-11.0)	8.0 (5.0-12.2)	.12
VA-LRTI, %			.26
None	266 (81.1)	148 (75.5)	
VAT	32 (9.8)	22 (11.2)	
VAP	30 (9.1)	26 (13.3)	

Abbreviations: AIDS, acquired immunodeficiency syndrome; ARDS, acute respiratory distress syndrome; COPD, chronic obstructive pulmonary disease; IQR, interquartile range; SAPS 2, Simplified Acute Physiology Score 2; SD, standard deviation; SOFA, Sequential Organ Failure Assessment; VA-LRTI, ventilator-associated lower respiratory tract infections; VAP, ventilator-associated pneumonia; VAT, ventilator-associated tracheobronchitis.

Results for multivariate logistic regression for ICU mortality are shown in Table 2. Neither VA-LRTI nor VAT or VAP individually was associated with worse outcome in both models. Bootstrap errors were small (Table 2). This means that if data were hypothetically replicated 10^5^ times, the results for the logistic regression would vary little between all possible data combinations. This suggests that the model is probably robust and was probably not overfitted.

**Table 2.** Logistic Regression Results for ICU Mortality.

Variable	OR	CI	*P*	Bootstrap SE	Bootstap CI
VA-LRTI model					
SAPS 2	1.02	1.01-1.03	<.001	0.005	1.01-1.03
Barthel index	0.99	0.98-1.00	.06	0.003	0.98-1.00
Worsening gas exchange	1.46	0.82-2.60	.19	0.306	0.79-2.65
VA-LRTI	1.07	0.62-1.83	.80	0.280	0.61-1.85
VAT/VAP model					
SAPS 2	1.02	1.01-1.03	<.001	0.005	1.01-1.03
Barthel index	0.99	0.98-1.00	.06	0.003	0.98-1.00
Worsening gas exchange	1.36	0.74-2.50	.31	0.325	0.71-2.56
VAT	0.94	0.49-1.77	.87	0.338	0.48-1.83
VAP	1.30	0.61-2.74	.49	0.399	0.59-2.84

Abbreviations: CI, confidence interval; ICU, intensive care unit; OR, odds ratio; SAPS 2, Simplified Acute Physiology Score 2; SE, standard error; VA-LRTI, ventilator-associated lower respiratory tract infections; VAP, ventilator-associated pneumonia; VAT, ventilator-associated tracheobronchitis.

In the whole TAVeM study, the attributable mortality of ARDS was 4.5% (95% confidence interval [CI]: 1.9%-7.2%) while attributable mortality for VA-LRTI was 3.2% (95% CI: 0.2%-6%). In the subgroup of patients without ARDS, attributable mortality for VA-LRTI was 7.4% (95% CI: 3.4%-11.4%); while in the subgroup of patients with ARDS, no attributable mortality for VA-LRTI was found (−16.6%; 95% CI: −44% to 10.9%). When only VAP was considered, its attributable mortality slightly increased in patients without ARDS (8.5%; 95% CI: 5.5%-11.6%) but remained nonsignificant in patients with ARDS.

**Figure 3.**
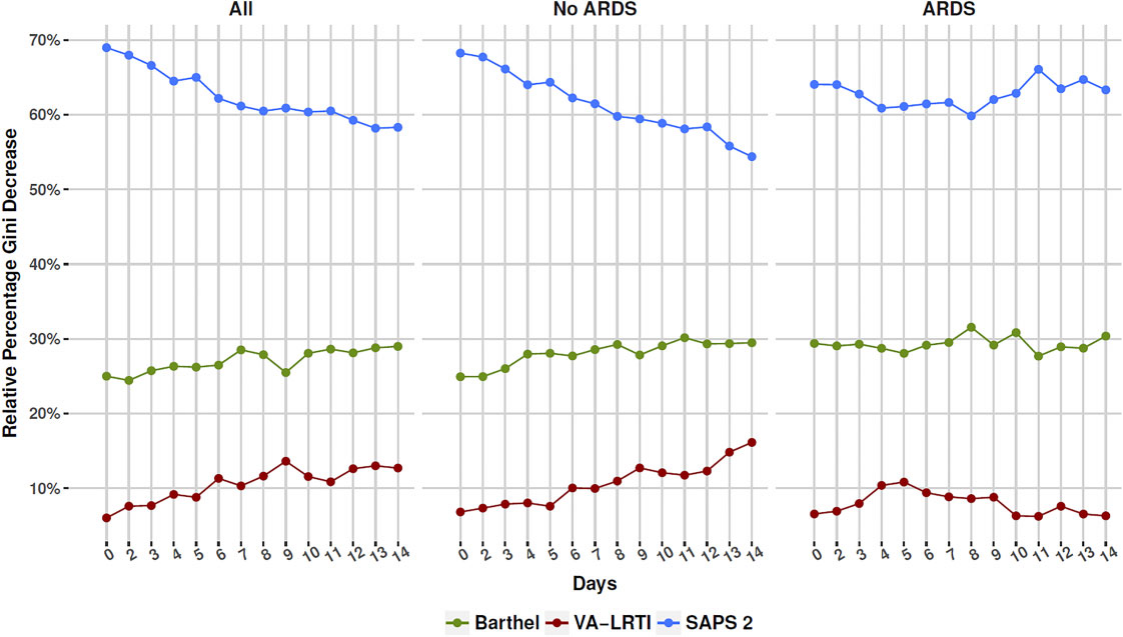
Relative variable importance for sequential random forest models performed from inclusion in the study and up to 14 days; panels (B) and (C) present the results for the non-acute respiratory distress syndrome (non-ARDS) and ARDS subgroups. Patients included in each model included those still alive in the intensive care unit (ICU) until the model reference day and which did not have ventilator-associated lower respiratory tract infection (VA-LRTI) until that moment. The relative importance was calculated as the mean Gini decrease for each variable divided over the total sum of Gini values for the model. Notice how the relative importance of Simplified Acute Physiology Score 2 (SAPS 2) decreased in patients without ARDS while the importance of Barthel index and VA-LRTI increased. For patients with ARDS, no clear trend was seen, with a questionable decrease in the importance of VA-LRTI over time.

The results of the sequential importance of variables in random forest models are shown in Figure 3. Simplified Acute Physiology Score 2 was the most important variable, followed by Barthel index and presence of VA-LRTI. In the whole population and in the non-ARDS subgroup, the relative importance of SAPS 2 decreased over time, while the importance of Barthel index and occurrence of VA-LRTI increased. In the ARDS population, however, relative importance fluctuated over time, with no suggestion of increase in the importance of VA-LRTI for the model over time. The relative Gini decrease over time for VA-LRTI remained below 10% during most of the time.

Although both ICU LOS and duration of MV were higher in patients with LRTI (Supplemental Figures 1 and 2), there was no significant association between both outcomes and VA-LRTI in competing risk analysis in patients with ARDS (hazard ratio [HR]: 0.82; 95% CI: 0.53-1.27, *P* = .38 and HR: 0.87, 95% CI: 0.58-1.30, *P* = .87 and *P* =.50).

## Discussion

Ventilator-associated lower respiratory tract infection has been a matter of debate, especially regarding its attributable mortality in specific clinical scenarios. This analysis of a large population that comprised almost 3000 patients from 114 ICUs in 8 countries found that the occurrence of VA-LRTI in patients with ARDS had no major impact in ICU mortality nor was it associated with increase in ICU LOS or duration of MV. Although the relative importance of VA-LRTI in a predictive model for mortality increased over time in patients without ARDS, it remained small (with a trend of decrease in importance) in patients with ARDS. This resulted in a global attributable mortality of VA-LRTI close to 3% but in a neglectable attributable mortality in the ARDS subgroup.

The most important finding in our analysis is the lack of association between VAP and mortality in patients with ARDS. This is something that needs to be analyzed cautiously because the most important risk factor for monitoring response to therapy in patients with VAP might be the improvement of oxygenation with either P/F ratio alone or in combination with other clinical parameters of the Clinical Pulmonary Infection Score.^16^ Therefore, suspicion for VA-LRTI could be triggered by a worse clinical scenario with persistently impaired, slowly resolving, or worsening oxygenation in patients with ARDS, which could lead to microbiological testing only in the setting of a worsening clinical scenario. Since our study was not designed to quantify the oxygenation impairment in the selected sample, we could not evaluate course in oxygenation improvement, which would be necessary to better understand our results. It should also be taken into account the inherent difficulties in diagnosing VA-LRTI in ARDS since these patients have bilateral radiographic infiltrates and present with worsening of oxygenation. In this context, diagnosis without microbiological confirmation may be unreliable. Ventilator-associated tracheobronchitis diagnosis may be even more cumbersome since VAT can only be assumed in the context of absence of new or progressive radiographic changes and demands tracheal aspirates for microbiologically confirmation.^17^ Either way, our results suggest that when VA-LRTI is suspected in patients with ARDS, the presence of positive cultures obtained from the respiratory tract coupled with clinical picture of respiratory infection is not associated with worse ICU mortality, regardless of radiographic findings (which may be subjective and discriminate between VAT and VAP).

One other important aspect is the trend in the variable importance over time for illness severity (SAPS 2), a proxy of baseline performance (Barthel index), and the occurrence of VA-LRTI. In patients without ARDS, the relative importance of a global illness severity marker as a mortality predictor decreased as early as after the first day of ICU admission, while Barthel index and occurrence of VA-LRTI increased their relative importance up to day 14. This finding is in accordance with other reports that suggested that baseline health status and comorbidities may become the major determinants of outcome early in the course of critical illness.^14^ In contrast, in patients with ARDS, the relative contribution of the 3 aforementioned variables was relatively constant; if any, there was a trend in the reduction of the already questionable importance of VA-LRTI during time. This may suggest that for the most severe critically ill patients, the baseline illness severity at admission is the major contributor to short-term outcome.

In one of the first multicenter studies of VA-LRTI in ARDS, Markowicz et al found results that are similar to ours.^7^ Microbiologically confirmed VAP occurred in 36.5% of the 134 patients with ARDS and was not associated with higher ICU mortality, although length of MV was longer when VAP occurred. In our analysis, when appropriate competing risks were applied, no effect of VA-LRTI on duration of MV or ICU LOS could be found. Several other small single-center studies assessed the impact of VAP in patients with ARDS, with varied results. More recently, Forel et al obtained similar results in patients ventilated according to a lung protective strategy.^8^ Using data from the PROSEVA study, Ayzac et al found that when VAP was treated as a time-dependent variable, it was associated with higher mortality in patients with ARDS.^4^ Our data exceed previous reports by considering both VAT and VAP in this population and by evaluating variable importance over time. Taken together, our findings suggest that in the population of severely ill patients with ARDS, microbiologically confirmed VA-LRTI may be more a marker than a reason for higher mortality.

Our analysis has several limitations. First, our sample size is reasonably small, which may have limited the number of confounders to be accounted for in regression models. The wide CIs highlight that our data are compatible with either an increase or decrease in mortality associated with VA-LRTI. We only had a very short-term end point (ICU mortality) available, which therefore hinders us from assessing whether VA-LRTI could contribute to mortality after ICU discharge. As we did not record specific interventions and process of care measures, we could not correct for many factors known to be associated with ARDS mortality such as inappropriate ventilatory settings, information on lung mechanics, and use of other treatments such as neuromuscular blockade. This may in part explain why we could not find specific variables associated with mortality in patients having ARDS with VA-LRTI. Second, the attributable mortality of ARDS was low in our population, which may be related to the overall low ARDS mortality in TAVeM population (37.4%); nevertheless, this value is close to some recent reports^18^ and may be related to the fact that even patients with mild ARDS were included in the analysis. No data on ARDS severity other than global severity of illness markers (including P/F ratio) were consistently available; therefore, we cannot determine whether these findings may be limited to patients with severe ARDS. We have also not assessed the impact of multiple events (ie, several episodes of VA-LRTI) on outcome. Finally, we only included microbiologically confirmed VA-LRTI; since the sensibility of respiratory track samples is not perfect, some patients with VA-LRTI may not have been included.

## Conclusion

Occurrence of VA-LRTI in patients with ARDS may not be associated with ICU mortality nor ICU LOS or duration of MV.

## Supplemental Material

Supplementary_File - Lower Respiratory Tract Infection and Short-Term Outcome in Patients With Acute Respiratory Distress Syndrome Click here for additional data file. Supplementary_File for Lower Respiratory Tract Infection and Short-Term Outcome in Patients With Acute Respiratory Distress Syndrome by Fernando G. Zampieri, Pedro Póvoa, Jorge I. Salluh, Alejandro Rodriguez, Sandrine Valade, José Andrade Gomes, Jean Reignier, Elena Molinos, Jordi Almirall, Nicolas Boussekey, Lorenzo Socias, Paula Ramirez, William N. Viana, Anahita Rouzé, Saad Nseir, Ignacio Martin-Loeches and on behalf of the TAVeM study group in Journal of Intensive Care Medicine
