# Supplementary material for: Lower Respiratory Tract Infection and Short-Term Outcome in Patients With Acute Respiratory Distress Syndrome
Source: J Intensive Care Med. 2018 Apr 26;35(6):588–94. doi: 10.1177/0885066618772498 (PMC7272129; doi:10.1177/0885066618772498)
Supplement: Supplementary_File - Lower Respiratory Tract Infection and Short-Term Outcome in Patients With Acute Respiratory Distress Syndrome [file Supplementary_File.pdf]

Lower respiratory tract infection and short-term outcome in patients with acute respiratory distress syndrome

Supplemental Electronic File

e-Table 1 – Comparison between Non-ARDS and ARDS patients

	Non-ARDS	ARDS	p
**Number of Patients**	2436	524	
**Age (mean ± SD))**	61.7 ± 16.9	61.7 ± 16.2	0.990
**Male Gender, n (%)**	1539 (63.2)	310 (59.2)	0.094
**Admission Type, n (%)**			<0.001
**Medical**	1484 (60.9)	404 (77.1)	
**Surgical**	453 (18.6)	91 (17.4)	
**Trauma**	499 (20.5)	29 (5.5)	
**SAPS2 (mean ± SD)**	50.6 ± 18.5	51.07 ± 19.0	0.616
**Barthel (mean ± SD)**	82.2 ± 31.2	83.9 ± 28.8	0.258
**SOFA (mean ± SD)**	7.9 ± 3.8	8.9 ± 3.9	<0.001
**COPD, n(%)**	423 (17.4)	71 (13.5)	0.039
**Chronic Renal Failure, n (%)**	220 (9.0)	73 (13.9)	1.000
**Diabetes, n (%)**	446 (18.3)	122 (23.3)	0.010
**Alcoholism, n (%)**	311 (12.8)	47 (9.0)	0.019
**Non-metastatic cancer, n (%)**	239 (9.8)	57 (10.9)	0.510
**Metastatic Cancer, n (%)**	97 (4.0)	15 (2.9)	0.275
**Hematologic Cancer, n (%)**	87 (3.6)	36 (6.9)	0.001
**AIDS, n (%)**	42 (1.7)	7 (1.3)	0.658
**Worsening X Ray, n (%)**	347 (14.2)	75 (14.3)	1.000
**Worsening Gas Exchange, n (%)**	383 (15.7)	90 (17.2)	0.449
**Time to Infection (median [IQR])**	6.0 [4.0, 10.0]	7.0 [4.0, 12.0]	0.011
**VA-LRTI (%)**			0.333
**None**	1857 (76.2)	414 (79.0)	
**VAT**	266 (10.9)	54 (10.3)	
**VAP**	313 (12.8)	56 (10.7)	
**Mortality, n (%)**	716 (29.4)	196 (37.4)	<0.001

e-Figure 1 – ICU Length-of-stay according to occurrence of VA-LRTI in patients with and without ARDS

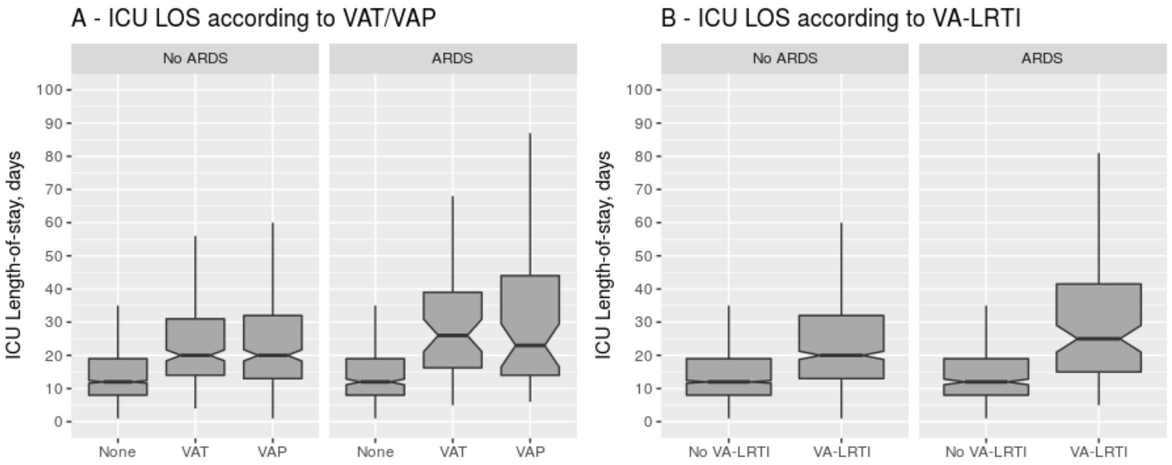

e-Figure 2 – Duration of mechanical ventilation according to occurrence of VA-LRTI in patients with and without ARDS

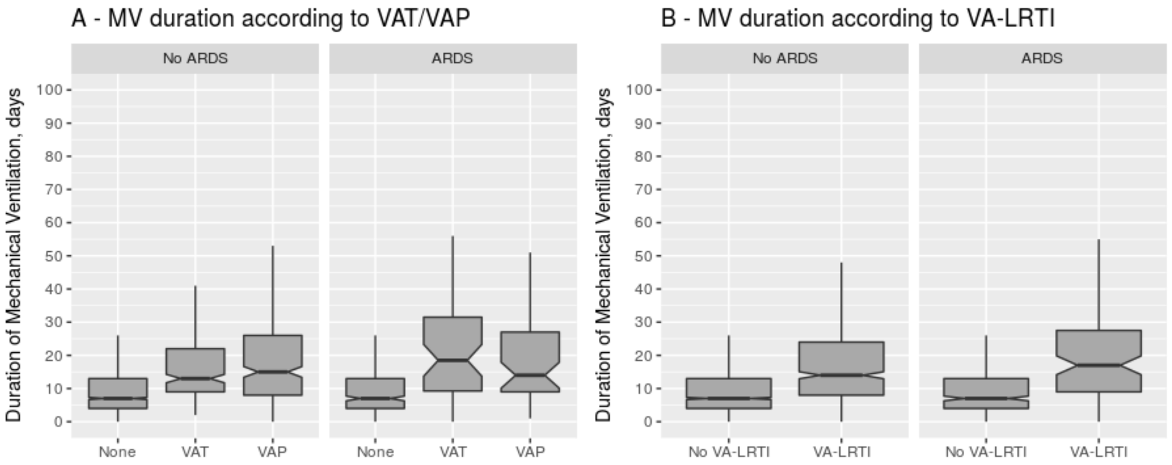

List of TAVeM Investigators

Ignacio Martín-Loeches, Alejandro Rodriguez, Daniel Curcio, Rubén Oscar Fernández, Jorge Arroyo, Maria Gabriela Rodriguez Alvarez, Alex Tamayo Reyes, Christian Dellera, Francisco Molina, Daniel Molano Franco, Edwin Giovanny Chapeta Parada, Estuardo Salgado Yepez, Fernando Paredes Oña, Diego Morocho Tutillo, Diego Barahona, Francisco Alvarez Lerma, Ana Abella Álvarez, Jose Manuel Allegue Gallego, Francisco José Fuentes Morillas, Antonio Luis Ruiz Aguilar, María Lourdes Cordero Lorenzana, Rafael Sanchez Iniesta, Jordi Almirall, Antonio Albaya, Sergio Ruiz Santana, Carmen Fernandez, Miguel Angel Blasco Naval Potro, Pablo Vidal Cortes, Belen Jimenez, Rafael Sierra, Maria Del Valle Ortiz, Nieves Cruza, Pedro Maria Olaechea, Ana Carolina Caballero Zirena, Pilar Posada Gonzalez, Teresa Recio Gomez, Lorenzo Socias Crespi, Paula Ramírez Galleymore, Ricard Jordà Marcos, César Palazón, Bernardo Gil Rueda, Juan Carlos Ballesteros, Maria Pilar Gracia Arnilla, Antonia Socias, Joaquin Amador, Esperanza Molero Silvero, Laura Macaya Redín, Mónica Zamora Elson, Luis Cabré Pericas, Joaquín Álvarez Rodríguez, Mercedes Nieto, Antoni Torres, Elena Molinos, Ana Josefina, Nuno Catorze, Pedro Póvoa, Carlos Candeias, Luis Coelho, Paulo André, Carolina Paulino, José Andrade, Gomes, Elisa Vedes, Antero Fernandes, Miguel ÁngelGarcía García, Catalina Sanchez Ramirez, Milagros Calizaya, Angel Estella, Adrià Albis, Gerardo Aguilar, Eva Torrents, Marta Gurpegui Puente, Angel Gabriel Sanchez, Thiago Lisboa, Pedro Azambuja, Marcos Freitas Knibel, Otavio Ranzani, Laura Darriba W. Camargo, Antonio Paulo Nassar Junior, Cesar Biselli Ferreira, Suzana Lobo, Ligia Rabello, Marcelo Park, Alexandre Guilherme Ribeiro de Carvalho, Mauricio Valencia, Alejandro Gonzalez Castro, Adoración Alcalá López, José María Castillo Caballero, Saad Nseir, Karim Jaffal, Erika Parmentier-Decrucq, Sébastien Préau, Chloé Rousselin, Caroline Blazejewski, Juliette Masse, Laurent Robriquet, Léa Satre-Buisson, Jean-Paul Mira, Nathalie Martin Raphael Lepecq, Hervé Mentec, Christophe Girault, Antoine Marchalot, Jonathan Messika, Jean-Damien Ricard, Philppe Seguin, Bruno Mégarbane, Sandrine Valade, Elie Azoulay, Nicolas Boussekey, Olivier Leroy, Jean Reignier, Marc Clavel, Nicolas Pichon, Thomas Baudry, Laurent Argaud, Pascal Beuret, Ali Ait-Hssain, Martine Nyunga, Isabelle Alves, Florent Dewavrin, Guillaume Brunin, Stéphane Mérat, Pierre Pasquier, Frédéric Brun, Aurore Palud, Benoit Voisin, Romaric Grenot, Nicolas Van Grunderbeeck, Didier Thévenin, Benoit Misset, François Philippart, Jean-Pierre Frat, Rémi Coudroy, Philippe Cabaret, Marie Ledein, Zoaire Slimane, Romain Miguel-Montanes, Nicolas Weiss, Francis Bolgert, Bernard Just.
